# Automated trichome counting in soybean using advanced image‐processing techniques

**DOI:** 10.1002/aps3.11375

**Published:** 2020-07-28

**Authors:** Seyed Vahid Mirnezami, Therin Young, Teshale Assefa, Shelby Prichard, Koushik Nagasubramanian, Kulbir Sandhu, Soumik Sarkar, Sriram Sundararajan, Matt E. O’Neal, Baskar Ganapathysubramanian, Arti Singh

**Affiliations:** ^1^ Department of Mechanical Engineering Iowa State University Ames Iowa USA; ^2^ Colaberry Inc. 200 Portland Street Boston Massachusetts 02114 USA; ^3^ Department of Agronomy Iowa State University Ames Iowa USA; ^4^ Department of Entomology Iowa State University Iowa USA

**Keywords:** image processing, imaging, insect feeding, soybean, trichome

## Abstract

**Premise:**

Trichomes are hair‐like appendages extending from the plant epidermis. They serve many important biotic roles, including interference with herbivore movement. Characterizing the number, density, and distribution of trichomes can provide valuable insights on plant response to insect infestation and define the extent of plant defense capability. Automated trichome counting would speed up this research but poses several challenges, primarily because of the variability in coloration and the high occlusion of the trichomes.

**Methods and Results:**

We developed a simplified method for image processing for automated and semi‐automated trichome counting. We illustrate this process using 30 leaves from 10 genotypes of soybean (*Glycine max*) differing in trichome abundance. We explored various heuristic image‐processing methods including thresholding and graph‐based algorithms to facilitate trichome counting. Of the two automated and two semi‐automated methods for trichome counting tested and with the help of regression analysis, the semi‐automated manually annotated trichome intersection curve method performed best, with an accuracy of close to 90% compared with the manually counted data.

**Conclusions:**

We address trichome counting challenges including occlusion by combining image processing with human intervention to propose a semi‐automated method for trichome quantification. This provides new opportunities for the rapid and automated identification and quantification of trichomes, which has applications in a wide variety of disciplines.

Trichomes are hair‐like appendages extending from the plant epidermis (Levin, 1973). They serve many important biotic roles for plants, including interference with herbivore movement, preventing herbivory, and reducing the area available for the attachment of insect eggs (Handley et al., 2005). In addition, trichomes play many other important roles, such as the protection of the plant from solar radiation, salt balance in the form of excretion, seed dispersal, and reduced evaporation (Ghorashy et al., 1971; Baldocchi et al., 1983; Serna and Martin, 2006; Price et al., 2011). There are many types of trichomes, including non‐glandular, glandular, curly, straight, unicellular, multicellular, hooked, and simple appendages, and they vary in their size and origin (Werker, 2000).

Glandular trichomes are different from non‐glandular structures because of the various substances they secrete, including sticky compounds and toxins. The sticky exudate from glandular trichomes can potentially entrap or completely immobilize insects that cross its path. Glandular trichomes may also release toxic substances that have multiple effects on insects, including reducing growth rates and preventing oviposition. This increases the mortality of the insect in comparison with individuals living on glabrous leaf varieties with no trichomes (Stipanovic, 1983). Non‐glandular trichomes lack the ability to produce sticky substances or toxins. Even without the presence of secondary substances, non‐glandular trichomes still act as an effective physical barrier against certain insects because of variations in trichome branching, length, and density (Levin, 1973; Stipanovic, 1983).

Soybean (*Glycine max* (L.) Merr.) produces long, dense trichomes to slow phloem‐feeding insects by preventing easy access to the plant surface itself (Price et al., 2011). Glabrous soybean pods were more damaged by the feeding of the adult bean leaf beetle (*Cerotoma trifurcata*) than pods with varying amounts of trichomes (Lam and Pedigo, 2001). The potato leaf hopper (*Empoasca fabae*) and springtails (*Deuterosminthurus yumanensis*) formed significantly larger populations on glabrous soybean varieties (Turnipseed, 1977). Significantly higher larval mortality and decreased pupal weight of Mexican bean beetle (*Epilachna varivestis*) were reported in soybean varieties with more trichomes (Gannon and Bach, 1996), and lower soybean looper (*Pseudoplusia includens*), jassid (*Amrasca biguttula*), and whitefly (*Bemisia tabaci*) infestation was reported in the presence of trichomes (Ihsan‐ul‐Haq et al., 2003). Dai et al. (2010) reported that soybean aphid (*Aphis glycines*) populations were similar across varieties with varying trichome densities; however, their study did not artificially infest plants with aphids nor limit exposure to the natural enemies of the aphid, thereby limiting the inference about the direct effect of trichomes on soybean aphids.

Trichomes, if effective in preventing insect infestation in soybean, could be a cost‐effective target for preventing crop losses; however, a major deterrent in advancing trichome studies is the tedious and time‐intensive phenotyping required to quantify the numbers of trichomes on the leaf surface. Few studies have used imaging‐based phenotyping to study soybean trichomes, with most approaches being predominantly manual (Kim et al., 2011; Cheng et al., 2014). Manual counting is both time consuming and mistake prone (Pomeranz et al., 2013); therefore, accurately identifying and counting soybean leaf trichomes is a serious bottleneck. Automating (or semi‐automating) the counting of trichomes could significantly advance research on trichomes; however, it is challenging because of the variability in trichome coloration and their high levels of occlusion. Due to its potential in automation and the reduced error associated with digital‐based phenotyping, image processing presents an exciting possibility to overcome the challenges associated with trichome phenotyping, expediting the counting process and potentially reducing the error associated with manual counting. The main objective of this study was therefore to explore and assess methods for determining trichome density in soybean leaves using image‐processing techniques.

## METHODS AND RESULTS

### Genetic materials

We used 10 isogenic soybean lines varying in trichome density (Table 1, Appendix S1). Two seeds of each of the 10 soybean genotypes were individually planted in soil substrate potting mix (Professional Growing Mix, Sun Gro Horticulture, Agawam, Massachusetts, USA) in 18‐cm pots. Around 0.5 g of Osmocote Plus 15‐9‐12 (Charleston, South Carolina, USA) was then added to each pot and lightly watered in. The plants were placed into a Percival E41L2C9 growth chamber (Percival Scientific, Perry, Iowa, USA). The growth chamber settings consisted of a 14 h : 10 h light : dark cycle with a constant temperature of 28°C and a relative humidity of 60%. Plants were checked daily and watered as needed.

**Table 1:** Ten isogenic lines with a shared parental background (Clark) with varying trichome densities were compared with two near‐isogenic (~75%) lines with or without recombination‐activating genes (RAGs).

Plant introduction ID	Trichome type/phenotype	RAG	RAG function	Reference
PI 548533	Clark	—	—	
PI 547410	Glabrous	P1	—	Pfeiffer, 1992
PI 547412	Glabrous	P1	—	Nagai and Saito, 1923; Hunt et al., 2011
PI 547415	Dense 1	Pd1	Controls the formation of a dense pubescence phenotype	Bernard and Singh, 1969; Komatsu et al., 2007; Palmer and Kilen, 1987
PI 547422	Sparse	Ps	Controls the formation of fewer trichomes per unit area	Hill et al., 2004; Komatsu et al., 2007; Palmer and Kilen, 1987
PI 547532	Sparse	Ps	Controls the formation of fewer trichomes per unit area	Bernard and Singh 1969; Komatsu et al., 2007; Palmer and Kilen, 1987
PI 547576	Sharp pubescence tip	Pb	Controls the formation of sharp trichome tips	Ting, 1946; Broich and Palmer, 1981
PI 547625	Dense 2	Pd2	Controls the formation of a dense pubescence phenotype	Palmer et al., 2004; Palmer and Kilen, 1987
PI 547643	Normal pubescence	Clark‐Rsv1	—	Ren et al., 2000
PI 547649	Extra‐dense	Pd1Pd2	Controls the formation of an extra‐dense pubescence phenotype	Bernard et al., 1991; Gunasinghe et al., 1988

When the soybean seedlings reached the vegetative stage with cotyledons (VC) (i.e., unrolled unifoliate leaves; Licht, 2014), one of the two plants from each pot was removed. When plants reached the V2 growth stage (i.e., two trifoliate leaflets unrolled), the leaves were imaged for the automated trichome count. The images were taken using a Nikon SMZ745T microscope (Nikon, Tokyo, Japan). The software used for the imaging was supplied with the microscope (Nikon Imaging Software) and was used to visualize the sample during imaging. All samples were put into focus using the highest magnification (7.5 : 1) so that the sample was in focus regardless of what magnification we decided to use for imaging. The tissue size used for imaging was approximately 25 mm × 25 mm.

### Leaf clearing and mounting

To better expose the trichomes during the imaging process, a chemical clearing process was used to remove the leaf chlorophyll and make the leaf transparent. Prior to the leaf‐clearing procedure, three trifoliate leaves of each of the 10 soybean genotypes were harvested and frozen for 24 h. The frozen leaves were allowed to thaw for one hour before beginning the clearing process. All clearing procedures were performed at room temperature under a fume hood.

For each of the 10 genotypes, one leaflet from each of the three trifoliate leaves was randomly chosen for imaging, for a total of 30 leaflets per genotype. The leaflets of each genotype were placed into beakers and submerged in denatured alcohol for 60 min to remove the epidermal wax. The residual alcohol was then poured off the leaves, after which they were soaked in a solution containing 100 mL of water and sodium hydroxide for 18 h to make them translucent. Soybean genotypes that appeared to have higher trichome densities were soaked for up to 24 h as these genotypes reached the translucent state at a slower rate. Once the leaves were translucent, they were placed in a Petri dish and rinsed three times with a gentle stream of water. The leaves were then neutralized with acetic acid for 30 min while still in the Petri dishes. The acetic acid was poured off the leaves, and sodium hypochlorite was added to the Petri dish for 30 min to clear the leaves. Afterward, the leaves were rinsed three times with water and placed on Kimwipes (Kimberly‐Clark, Irving, Texas, USA) so that the residual moisture could be absorbed. The leaves were left in covered Petri dishes at room temperature and set aside for the mounting procedure.

We utilized two leaf‐mounting techniques to capture the images. For *trichome density mounting*, a single tissue sample (~1 cm^2^) was cut from each of the three leaves for each genotype and placed on a single glass slide, so that there were three samples on one glass slide for each genotype. A second glass slide was then used to cover the three leaf samples and ensure that the leaves were flat and on the same viewing plane for imaging. The glass slides were held together with two strips of double‐sided tape, one at each end of the slide.

For *full‐length trichome mounting*, a single sample from each of the previously cut leaves of each genotype was cut and folded once so that trichomes on the bottom of the leaf were exposed and trichomes along the crease of the fold stood perpendicular to the crease. The leaves were not folded along the major veins as this region contained significantly more trichomes than other areas of the leaf and was not representative of the leaf area. The folded leaf samples were placed between two glass slides as in the trichome density mounting procedure.

### Trichome imaging

A Nikon SMZ 745T stereomicroscope equipped with a polarizing filter attachment and Infinity image analysis software (Teledyne Lumenera, Ottawa, Ontario, Canada) was used to capture the images of the soybean leaf trichomes. The mounted leaf samples were placed on the microscope sample stage, and images were captured after the desired lighting, magnification, and focus were achieved. The images did not include areas where major veins were visible. Adaxial and abaxial trichome density images were captured from the same samples because the transparent glass slides could easily be flipped to gain access to the opposite side of the mounted leaves. Images of the standing trichomes were captured from the mounted folded leaf samples. Side view images were used to count the number of trichomes (Appendix S2). For the adaxial and abaxial views, it was very difficult to separate out individual trichomes because of the significant occlusion and merging of trichomes when they overlap on each other. Instead, we visually determined whether the trichomes were absent, or of low, medium, or high density (Appendix S3). These differences correspond to the known variation in trichome density attributed to the isogenic set of genotypes used in this study.

### Trichome image preprocessing

#### Manual counting

All genotypes were manually phenotyped (counted). We only counted trichomes emerging from the leaf base. Trichomes that emerged from the left or right border of the image were not counted (Appendix S4).

#### Trichome segmentation and skeletonization

The image processing (Kulkarni and Patil, 2012) toolbox in MATLAB (MathWorks, Natick, Massachusetts, USA) was used to process and analyze the images. This toolbox has previously been used for other phenotyping tasks (Zhou et al., 2019). The pre‐processing steps included converting the images from RGB to the LAB color space, binarizing the images using a threshold value, and then skeletonizing the obtained images. This was done to enable the function of the image‐processing algorithm.

**Figure 1:**
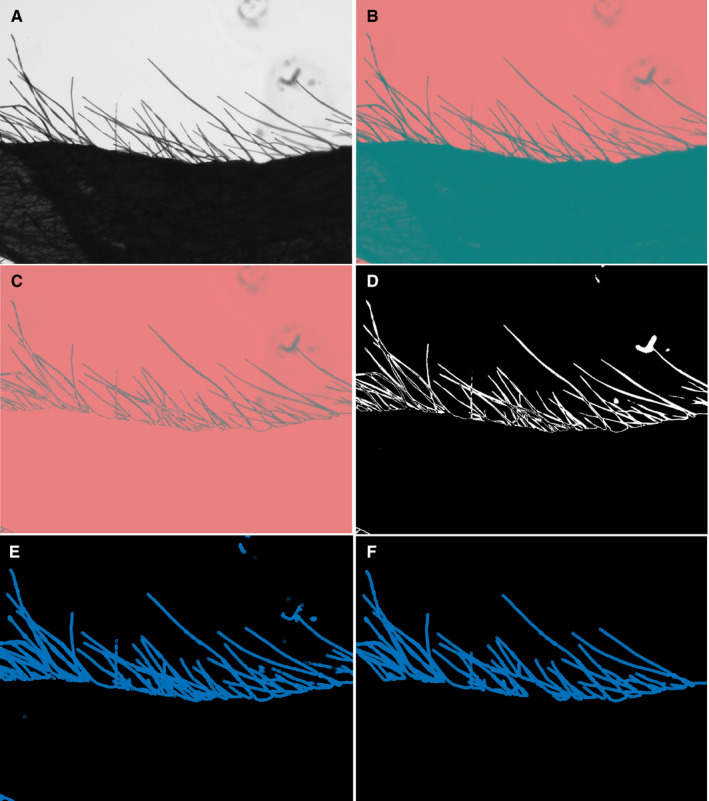
Image pre‐processing steps. The images were converted from (A) the RGB to (B) the LAB color space. (C) The leaf is then segmented. (D) Next, a binary image was obtained using Otsu thresholding. (E) A skeleton image was obtained. (F) Final processed image after thinning and selecting the biggest component to remove the noise artifacts.

Segmenting the trichomes in each image was the first step in all of the trichome counting methods outlined below. To do so, all images were converted from the RGB color space to the LAB color space. LAB is a color space with three axes: L refers to lightness while A and B are related to color dimension (Fig. 1). Because the leaf and trichomes are connected, a thresholding value was selected to separate the individual trichomes. The threshold value was applied over the lightness axis; hence, this color space is useful for trichome segmentation from the leaf. This value was heuristically obtained by comparing the pixel intensity of the light dimension of the LAB color space throughout the image. Figures 1A and 1B show the conversion between these two color spaces. In addition, Fig. 1C shows the bottom part of the image where the leaf surface was successfully distinguished from the trichomes. This image was converted from the LAB color space to grayscale for further analysis. Next, based on Otsu’s thresholding (Otsu, 1979), all images were binarized so that the trichomes were shown as white pixels. Next, skeletonization (using thinning methodology) was applied to represent each trichome as a line (Lam et al., 1992). These types of segmentation and skeletonization are widely used in plant phenotyping (Ma et al., 2019; Falk et al., 2020). We then applied a Gaussian filter to obtain a skeletonization with less noise. Additionally, noise removal was accomplished using the largest connected component algorithm. The skeleton image and the image obtained after noise removal are illustrated in Figs. 1E and 1F, respectively.

### Trichome counting methods

**Figure 2:**
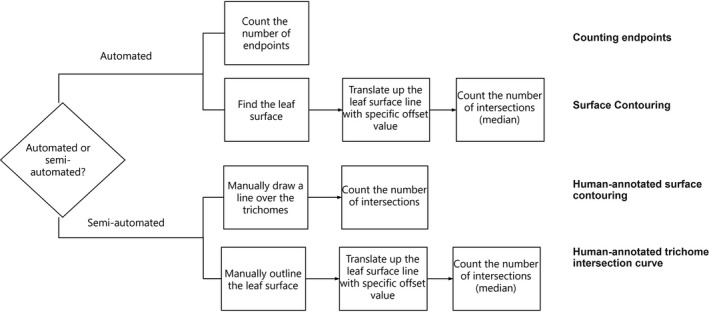
Flowchart of the different methods used to count the number of trichomes.

After the skeletonization step was performed, we explored both automated and semi‐automated methods to count the number of trichomes on the leaves. We considered two fully automated methods and two semi‐automated methods for counting the trichomes. The correlations between manual counting and each different counting method were compared using *R* ^2^. We outline the conceptual approach of these methods in Fig. 2.

#### Fully automated counting

##### Counting endpoints

In a skeletonized image, all trichomes are represented as line segments with two endpoints. One endpoint is on the surface of the leaf and the other endpoint is free. Thus, the number of free endpoints in the skeleton image gives a measure of the number of trichomes. This method can roughly estimate how dense the trichomes are.

**Figure 3:**
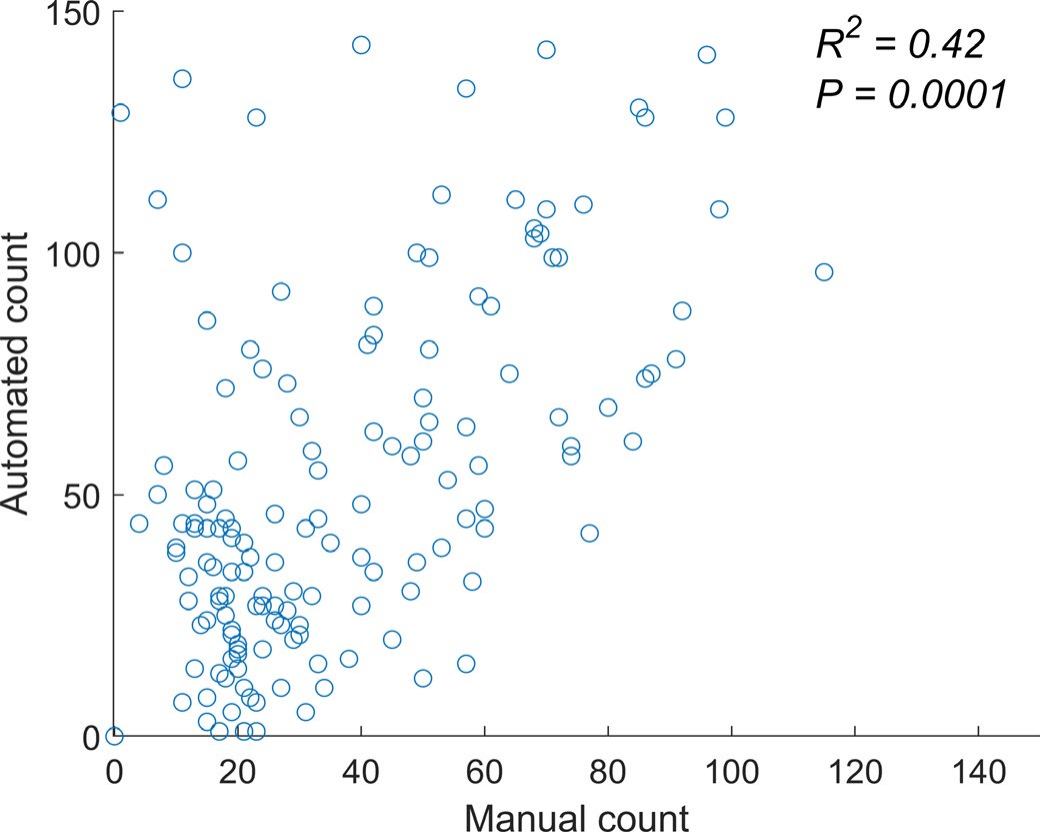
Comparison of the number of trichomes counted automatically and manually. *R* ^2^ value for the fully automated method is 0.42 (*P* = 0.0001) when compared with the actual number of trichomes from the manual count.

Figure 3 shows the number of trichomes detected using the counting endpoints method compared with results from the manual counting of the same images. We found that this method was quite sensitive to the presence of noise in the skeletonized image, which resulted in the overestimation of the number of trichomes. The average number of trichomes counted manually was 32.3, while for the automated counting it was 45.5; the root mean square error was 20, and the *R* ^2^ was moderate (0.42, *P* = 0. 0001).

The counting endpoints method was particularly effective when the trichome density was low, resulting in sparse images. When the trichome density was high, this method underperformed because of the impact of noise and occlusions. In the latter case, the end of a trichome may lie on the “stalk” of another trichome, meaning no endpoint was detected for that particular trichome. In addition, the presence of small hairs and protuberances due to noise were incorrectly counted as endpoints. One approach to circumvent these problems in cases of high trichome density was to apply a low pass filter to blur (and denoise) the skeleton image.

##### Automated surface contouring

**Figure 4:**
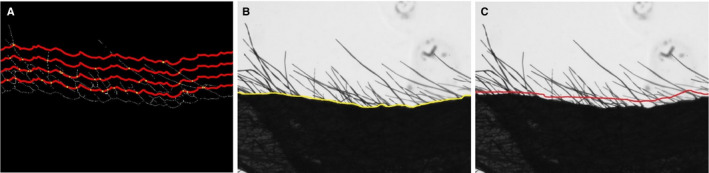
Surface contouring methods. (A) Four parallel curves drawn based on the leaf surface line and the offset value. (B) Human‐annotated surface contouring shown as a yellow line on the surface of the leaf. (C) Human‐annotated trichome intersection curve shown as a red line on the surface of the leaf.

In the second automated counting method, we circumvented the shortcoming of the endpoint counting approach by counting the number of trichome “stalks.” We first automatically detected the base curve of the leaf surface. This base curve was then translated vertically upward (see Fig. 4A). The number of intersections of the skeleton with the translated base curve provides an estimate of the number of trichomes at that height. We report the mean number of intersections across several vertical translations.

The base curve was detected by identifying the lowest white pixel in each column of the skeleton image. Four parallel curves with equal offset were then plotted (Fig. 4A), and the number of intersections between each curve and the skeleton images was subsequently calculated. The mean value of all intersections was reported as a measure of the number of trichomes. The offset value was decided based on the distance between the topmost and bottom‐most parts of the image where white pixels are located. The average trichome was divided into four intervals.

**Figure 5:**
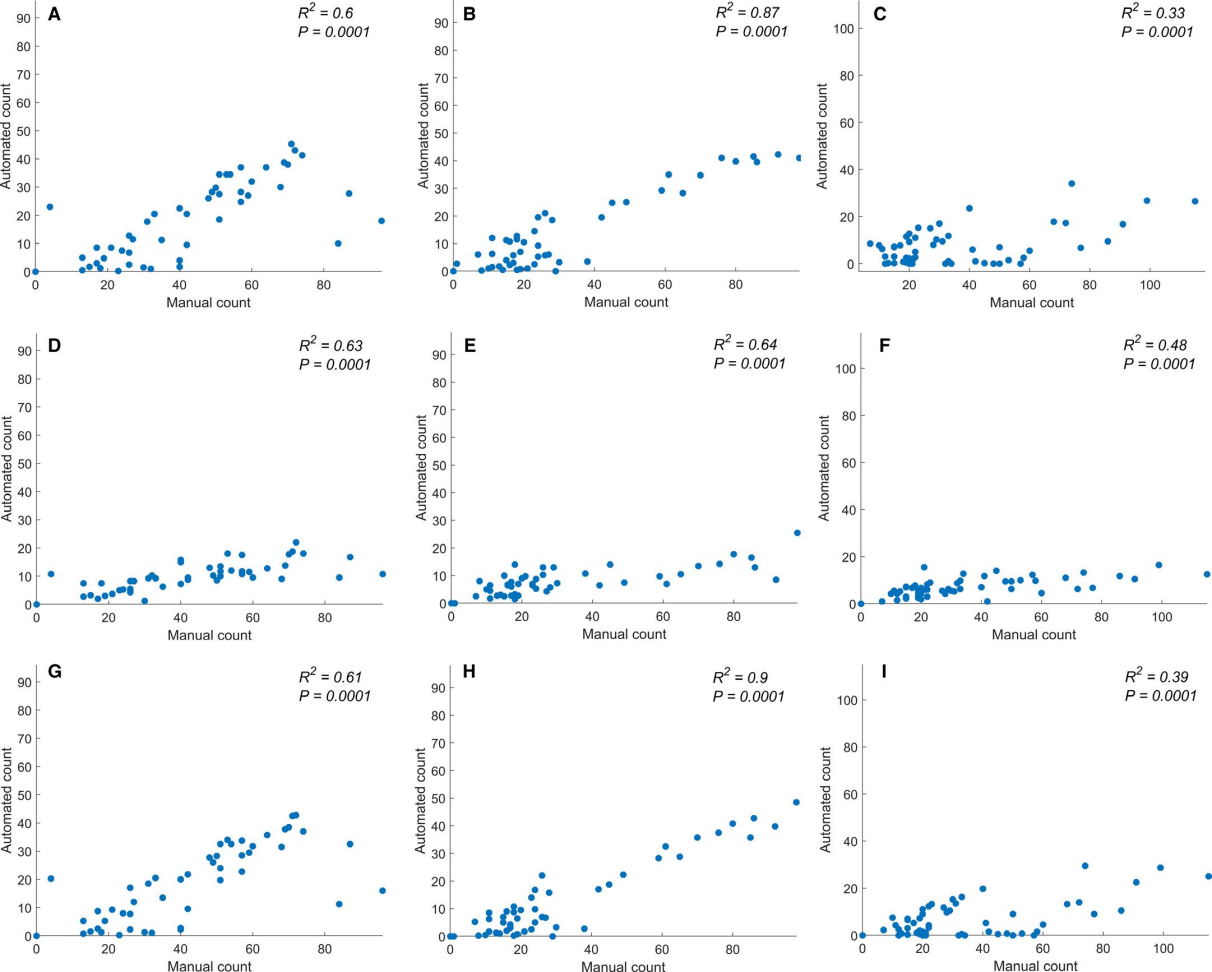
Comparison of the resulting number of trichomes obtained using each method for each repetition. (A–C) *R* ^2^ for repetition 1 (A) , repetition 2 (B), and repetition 3 (C) of the fully automated method using an automatically determined leaf surface line. The predicted data were compared with the ground truth. (D–F) *R* ^2^ for repetition 1 (D), repetition 2 (E), and repetition 3 (F) of the semi‐automated method performed using a manually drawn leaf surface line. The predicted data were compared with the ground truth. (G–I) *R* ^2^ for repetition 1 (G), repetition 2 (H), and repetition 3 (I) of the semi‐automated method performed using a manually drawn curve over the trichomes. The predicted data were compared with the ground truth.

The correlations between the results of manual counting and the automated surface contouring approach for each repetition are plotted in Fig. 5. The number of trichomes counted by this method was lower than for the manual counting; however, the *R* ^2^ for the first and second repetitions were relatively good (0.6 and 0.87, respectively). For the third repetition, the *R* ^2^ was only 0.33 (*P* = 0. 0001). The poor performance in the third repetition was due to the incorrect detection of the base leaf surface resulting from occlusion and the presence of strange shapes resulting from lack of evenness of the leaf surface. Blurring and skeletonization did not dramatically improve this issue. A shortcoming of this method is therefore that the accuracy of the results is determined to a large extent by the quality of the base curve.

#### Semi‐automated counting

##### Human‐annotated surface contouring

In the first semi‐automated method, we ensured the quality of the base curve by manually drawing a yellow curve such that it could pass through the base of all trichomes, as shown in Fig. 4B. This was done to eliminate the problem noted above for the third repetition and to create an accurate base curve, and was the only manual step in the process. Following this step, we automatically moved the base curve and counted the number of intersections with the skeletonized image. The average number of intersections between the translated base line and the skeleton image was calculated and reported as the number of trichomes.

We see a more consistent performance for the trichome count when using a human‐annotated base surface. Figure 5B shows a comparison of the number of trichomes obtained by this method versus the manual counting method. The *R* ^2^ values for the first and second repetitions were still substantial (0.63 and 0.64, respectively, with *P* values of 0.0001 and 0.0001); however, using this approach, the issue noted above for the third repetition in the automated method was now mostly resolved (*R* ^2^ = 0.48, *P* = 0.0001). The images in the third repetition were also denser than the other repetitions.

##### Human‐annotated trichome intersection curve

In the previous methods, the key idea was to translate the manually or automatically detected surface curve to identify the intersections of trichomes with the curve. In the final semi‐automated approach, the human annotator drew a single curve that passes through most of the trichomes. Figure 4C shows this (red) curve drawn by a human so that almost all the trichomes pass through this curve. Subsequently, the number of intersection points between this one curve and the skeleton image was reported as a measure of the number of trichomes.

The comparison between the manually counted trichomes and the number calculated by this method is shown in Fig. 5C. In this case, the *R* ^2^ values for the first and second repetitions were 0.62 and 0.90, respectively. This consistent performance is further improved by the manual annotation of a surface with a high number of trichome intersections.

## CONCLUSIONS

Characterizing the number, density, and distribution of trichomes can provide valuable insights into the stress responses of some plant species. In this work, we detailed a step‐by‐step approach to automatically or semi‐automatically quantify the number of trichomes on soybean leaves. Fully automating the estimation of trichome numbers is challenging as there are several difficulties to overcome, primarily because of the variability in coloration and the high occlusion of the trichomes. The quality of imaging (shadowing and contrast) affects the segmentation pipeline that isolates the foreground (trichomes) from the background.

Here, we showed that fully automated image‐processing approaches for trichome counting do not perform as well as methods that include some user input. Given that automated trichome counting is a difficult problem, we advocate for the deployment of machine learning algorithms (especially object‐detection and crowd‐sourcing algorithms) as promising techniques for future work, as these algorithms can have a more robust performance for cluttered images than image processing methods (Akintayo et al., 2018). However, images of sufficient quality and with varying trichomes density will be needed.

## AUTHOR CONTRIBUTIONS

A.S., B.G, S. Sundararajan, and S. Sarkar formulated the research problem and designed the approaches. S.V.M., T.Y., T.A., S.P., M.O., and A.S. collected the data. S.V.M., T.J., K.N., S. Sarkar, B.G., S. Sundararajan, and A.S. developed the processing workflow and performed the data analytics. All authors contributed to the writing and development of the manuscript. All authors read and approved the final manuscript.

## Supporting information


**APPENDIX S1.** Leaf surface phenotypes of the 10 soybean (*Glycine max*) isolines used in this study. Images taken at growth stage V2, i.e., plants with two sets of unfolded trifoliate leaves. Scale bar = 1 mm.

**APPENDIX S2.** The three different views of imaging for a single soybean genotype. Side view images were used to count the number of trichomes. For the adaxial and abaxial views, it was very difficult to separate out individual trichomes due to the significant occlusion and merging.

**APPENDIX S3.** Observed trichome density variation in the 10 isogenic soybean lines analyzed.

**APPENDIX S4.** Definition of trichome counting. (A) Trichomes were only counted when they were visibly emerging from the leaf base on the image (PI 547415, repetition 2). (B) Trichomes that emerged from the left or right border of the image were not counted (PI 547412, repetition 3).
