# Supplementary material for: Automated trichome counting in soybean using advanced image‐processing techniques
Source: Appl Plant Sci. 2020 Jul 28;8(7):e11375. doi: 10.1002/aps3.11375 (PMC7394713; doi:10.1002/aps3.11375)

DOI 10.1002/aps3.11375

APPENDIX S1. Leaf surface phenotypes of the 10 soybean (*Glycine max*) isolines used in this study. Images taken at growth stage V2, i.e., plants with two sets of unfolded trifoliate leaves. Scale bar = 1 mm.

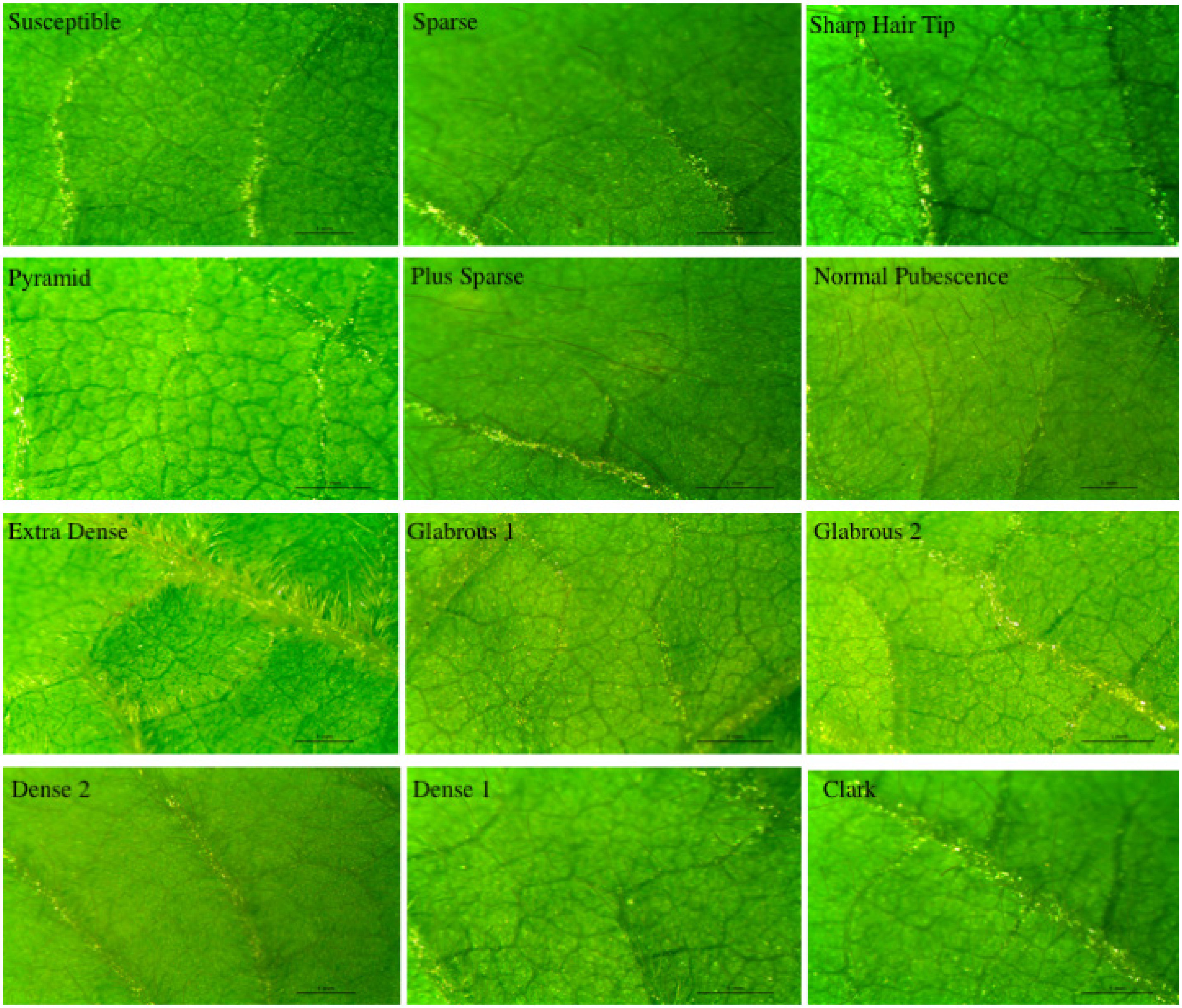

Supplement: Supplementary file 1 — APPENDIX S1. Leaf surface phenotypes of the 10 soybean (Glycine max) isolines used in this study. Images taken at growth stage V2, i.e., plants with two sets of unfolded trifoliate leaves. Scale bar = 1 mm. [file APS3-8-e11375-s001.pdf]
