# Supplementary material for: Automated trichome counting in soybean using advanced image‐processing techniques
Source: Appl Plant Sci. 2020 Jul 28;8(7):e11375. doi: 10.1002/aps3.11375 (PMC7394713; doi:10.1002/aps3.11375)

DOI 10.1002/aps3.11375

APPENDIX S2. The three different views of imaging for a single soybean genotype. Side view images were used to count the number of trichomes. For the adaxial and abaxial views, it was very difficult to separate out individual trichomes due to the significant occlusion and merging.

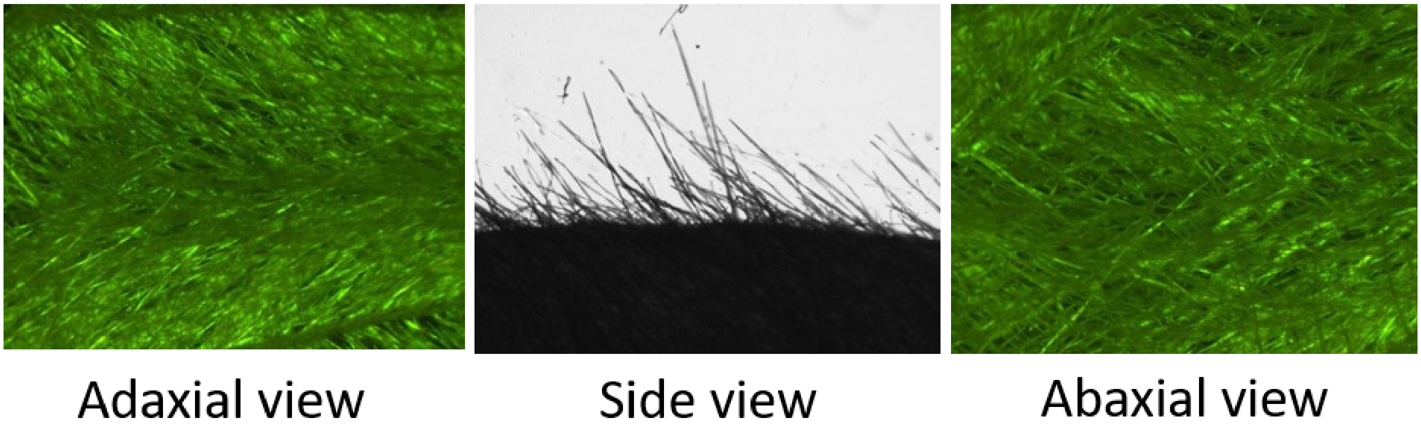

Supplement: Supplementary file 2 — APPENDIX S2. The three different views of imaging for a single soybean genotype. Side view images were used to count the number of trichomes. For the adaxial and abaxial views, it was very difficult to separate out individual trichomes due to the significant occlusion and merging. [file APS3-8-e11375-s002.pdf]
