# Supplementary material for: Automated trichome counting in soybean using advanced image‐processing techniques
Source: Appl Plant Sci. 2020 Jul 28;8(7):e11375. doi: 10.1002/aps3.11375 (PMC7394713; doi:10.1002/aps3.11375)
Supplement: Supplementary file 3 — APPENDIX S3. Observed trichome density variation in the 10 isogenic soybean lines analyzed. [file APS3-8-e11375-s003.pdf]

DOI 10.1002/aps3.11375

APPENDIX S3. Observed trichome density variation in the 10 isogenic soybean lines analyzed.

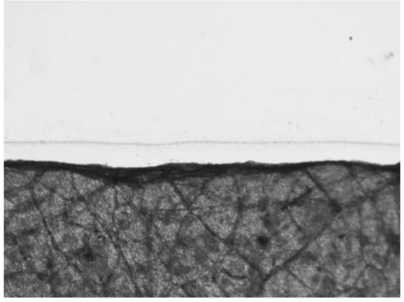

PI 547410

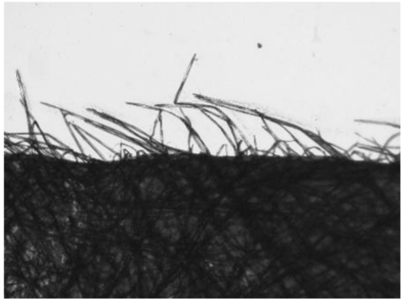

PI 547412

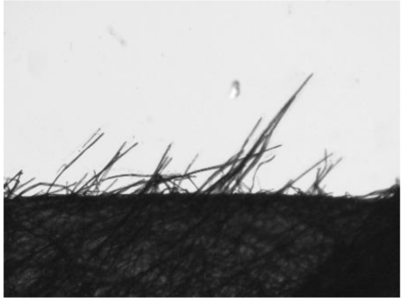

PI 547415

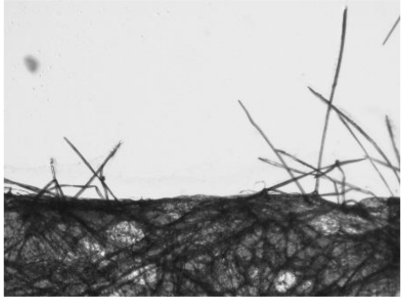

PI 547422

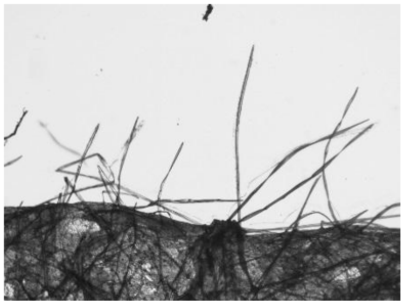

PI 547532

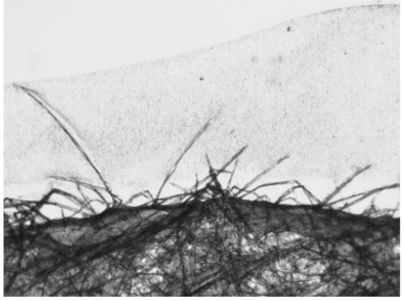

PI 547576

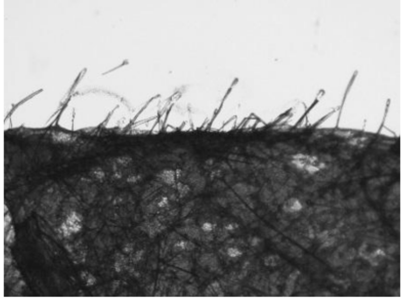

PI 547625

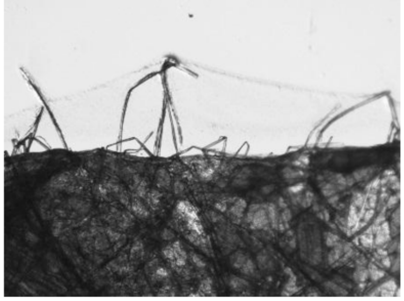

PI 547643

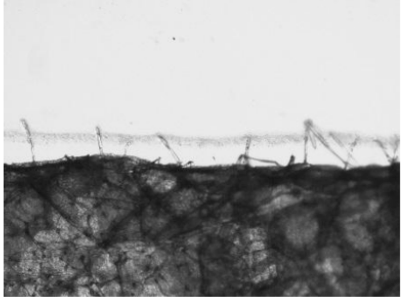

PI 547649

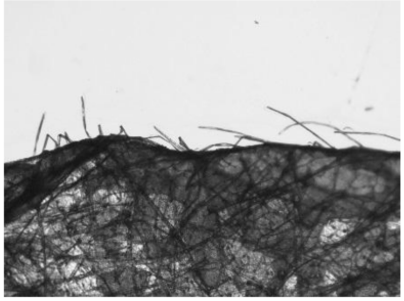

PI 548533
