# Supplementary material for: Automated trichome counting in soybean using advanced image‐processing techniques
Source: Appl Plant Sci. 2020 Jul 28;8(7):e11375. doi: 10.1002/aps3.11375 (PMC7394713; doi:10.1002/aps3.11375)

DOI 10.1002/aps3.11375

APPENDIX S4. Definition of trichome counting. (A) Trichomes were only counted when they were visibly emerging from the leaf base on the image (PI 547415, repetition 2). (B) Trichomes that emerged from the left or right border of the image were not counted (PI 547412, repetition 3).

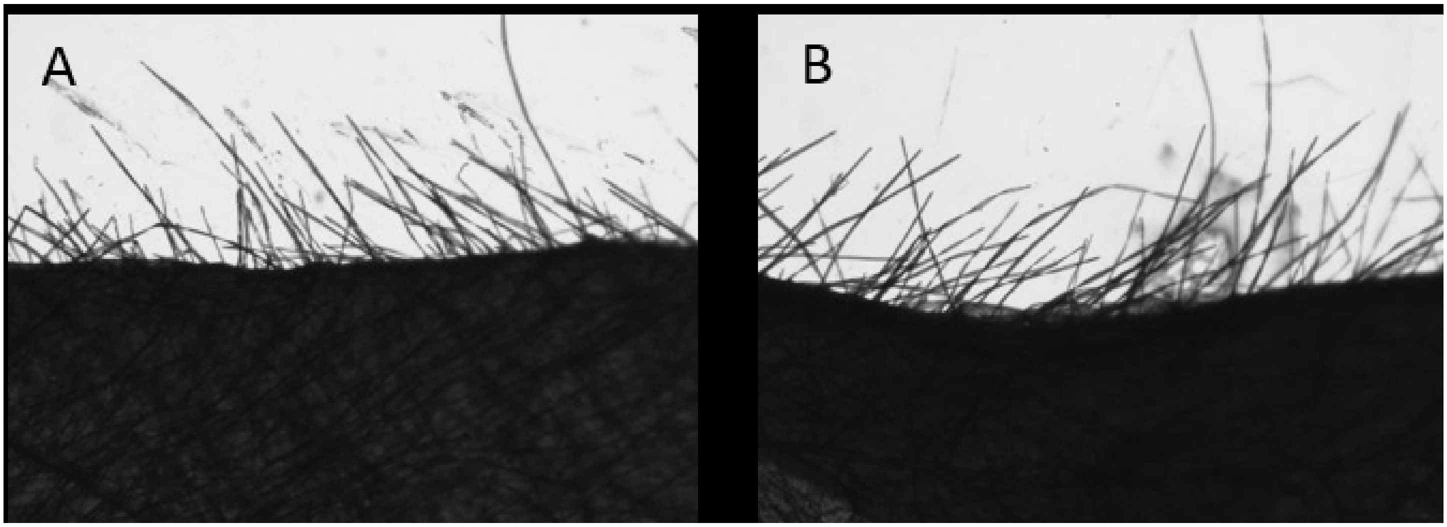

Supplement: Supplementary file 4 — APPENDIX S4. Definition of trichome counting. (A) Trichomes were only counted when they were visibly emerging from the leaf base on the image (PI 547415, repetition 2). (B) Trichomes that emerged from the left or right border of the image were not counted (PI 547412, repetition 3). [file APS3-8-e11375-s004.pdf]
